# Metacognition in Schizophrenia Spectrum Disorders—Current Methods and Approaches

**DOI:** 10.3390/brainsci13071004

**Published:** 2023-06-28

**Authors:** Petru Fabian Lungu, Corina-Miruna Lungu, Alin Ciobîcă, Ioana Miruna Balmus, Alexandra Boloș, Romeo Dobrin, Alina Costina Luca

**Affiliations:** 1 Faculty of Biology, Biology Department, “Alexandru Ioan Cuza” University, 700506 Iasi, Romania; lungufabian123@gmail.com (P.F.L.);; 2 Faculty of Psychology and Educational Sciences/Psychology Department, “Alexandru Ioan Cuza” University, 700506 Iasi, Romania; 3 Biomedical Research Center, Romanian Academy, 700506 Iasi, Romania; 4 Academy of Romanian Scientists, 010071 Bucharest, Romania; 5 Institute of Interdisciplinary Research, Department of Exact Sciences and Natural Sciences, “Alexandru Ioan Cuza” University, 700506 Iasi, Romania; 6 Departament of Psychiatry, “Grigore T. Popa”, University of Medicine and Pharmacy, 700115 Iasi, Romania; romeodobrin2002@gmail.com; 7 Department of Mother and Child Medicine-Pediatrics, “Grigore T. Popa”, University of Medicine and Pharmacy, 700115 Iasi, Romania

**Keywords:** schizophrenia, metacognition, measurement, tools

## Abstract

Metacognition essentially represents “thinking about thinking”, or the individual’s capacity to control and monitor their own cognitive processes. Metacognition impairment in schizophrenia represents a core feature of the disease, and, in the last fifteen years, the subject has evolved into a growing study area concentrating on a wide variety of processes, such as clinical insight, autobiographical memory, cognitive beliefs, reasoning, and memory biases. Since metacognition is a complex subject, we wanted to focus on the different nuances of metacognition transposed into the lives of patients diagnosed with either schizophrenia or a schizoaffective disorder. Therefore, this narrative review aims to analyze the literature in order to provide an insight regarding the current methods and approaches in the study of metacognition in schizophrenia or schizoaffective disorders, as well as the results provided. Results from the reviewed studies showed that patients with schizophrenia have a lower metacognitive ability, which is strongly reflected in their lives. Studies to date have highlighted the interaction between schizophrenia symptoms and metacognition, which shows how metacognition impacts work performance, autobiographical memory, motivation, the severity of symptoms, and social cognition.

## 1. Introduction

Schizophrenia disorders, according to the International Classification of Disorders-10th edition, are defined by general and specific thinking or perceptual distortions as well as blunted affect. Clear consciousness as well as intellectual abilities are typically preserved, although some cognitive deficiencies may evolve in the course of time. Of all schizophrenia spectrum disorders, paranoid schizophrenia and schizoaffective disorders are the most studied [1].

Paranoid schizophrenia is characterized by generally stable, often paranoid delusions, which are frequently accompanied by hallucinations, particularly auditory, and perceptual difficulties. Disturbances in mood, volition, and speech, as well as catatonic signs, are either absent or minor [1].

Schizoaffective disorders are disorders in which both affective and schizophrenic symptoms are prominent but do not justify a diagnosis of either schizophrenia or depressive or manic episode [1].

Schizophrenia affects 1 in 300 individuals worldwide, or about 24 million people globally. The current data show that 1 in 222 people, amongst adults, is affected [2]. There is no specific cause of schizophrenia that has been found by researchers. However, a series of factors of a genetic, environmental, or chemical nature can play an essential role in the occurrence of schizophrenia [3,4,5,6]. For example, season of birth has been connected to schizophrenia occurrence, including late winter/early spring in some areas and summer for the deficit type of the condition. Children growing up in cities, as well as some minority ethnic groups, have a greater prevalence of schizophrenia and associated diseases. Although most people with schizophrenia have no family history of psychosis, genetic factors do play a significant role in predicting risk for schizophrenia. Liability is conferred by a range of risk alleles, both common and rare, each of them contributing just a small percentage to the total population variance [3]. To date, risk alleles have been linked to a variety of mental diseases, including bipolar disorder, depression, and autism spectrum disorders. Pregnancy and delivery problems related to hypoxia and older paternal age are linked to an increased risk of schizophrenia in the developing baby. Stress, illness, malnutrition, maternal diabetes, and other medical disorders throughout pregnancy and the postnatal period have also been associated with schizophrenia. The great majority of infants with these risk factors, however, do not, most of the time, go on to be diagnosed with schizophrenia [3].

Metacognition is the higher-order thinking that involves active control of the cognitive processes involved in learning [7]. Research has indicated that many people with schizophrenia spectrum disorders experience metacognitive deficits such as impairments in social cognition, working memory deficits, processing speed, and visual and verbal learning dysfunctions, as well as significant deficits in reasoning, planning, abstract thinking, and problem solving [8]. Social cognition is also compromised, which is the capacity to appropriately incorporate information and apply it to develop adequate responses in certain circumstances. Cognitive deficiency and functional outcome are closely linked because patients with schizophrenia have affected metacognition and may be expected to have worse results in terms of metacognitive performance. They have difficulties forming complex ideas about self and others, show many memory biases, are not generally aware of their condition, and exhibit a significant impairment in their total cognitive ability. Since metacognition is important for learning and training, it is essential to investigate metacognitive activity and metacognitive evolution in order to identify how individuals with neurodegenerative diseases could be taught to use cognitive resources through metacognitive control [7,8].

Therefore, this narrative review aims to analyze the literature in order not only to observe the progress made in the last 15 years regarding the study of the various subcomponents of metacognition in patients with schizophrenia, but also to establish the role that metacognition has in this disorder.

## 2. Methods

We conducted a search for metacognition deficits in patients with schizophrenia, various ways those deficits were assessed, and the results obtained from the evaluation. For the most part, information was taken from the Medline, Elsevier, and Scopus databases and handpicked scientific journals published between 2009 and 2023 about the different strategies and methods used in the assessment of metacognition. The search keywords used were “metacognition and learning in schizophrenia”; “self-awareness in schizophrenia”; “metacognition and learning in delusions of schizophrenia”; “metacognitive profiles in schizophrenia”; “metacognition assessment in schizophrenia”; “metacognition and self-reflection in schizophrenia” “metamemory in schizophrenia” “social metacognition in schizophrenia” “work performance and metacognition in schizophrenia”, and “delusions in metacognition in schizophrenia”.

In order to refine the number of studies encountered during our search, we applied the following criteria:

1. Papers originally published in English. Titles and abstracts were screened for initial study inclusion. If the text of abstracts were promising, the full text was thoroughly examined to determine aims, materials and methods, and the results obtained.
2. Studies usually included participants that were diagnosed with a schizophrenia spectrum disorder (mainly paranoid schizophrenia or a schizoaffective disorder). We also considered studies that included patients with schizophrenia spectrum disorder in their cohort and the comparisons that were made between those patients and healthy controls or other psychiatric conditions (such as obsessive–compulsive disorder, bipolar disorder and posttraumatic stress disorder). Patients were in stable condition and had not changed medication in the last month.
3. The various components of metacognition were included, and comparisons were made between metacognition and deficiencies suffered by patients with schizophrenia (cognitive, affective, or behavioral), which could interact with metacognition.
4. Cohorts necessary with modest number of individuals but not less than 20 participants.

Since the number of studies that analyzed the interaction between metacognition and impairments suffered by participants diagnosed with a schizophrenia spectrum disorder was vast, we decided to use only those studies that approached interactions between metacognition and social cognition, motivation, work performance, metamemory, anxiety, empathy hallucinations, and delusions.

## 3. Results

Alongside the positive and negative symptoms, people with schizophrenia spectrum disorders often experience a decrease in self-awareness. Many individuals with schizophrenia have, for example, a lack of insight about their psychiatric condition [9,10], are unsure of the origin of things they have felt, thought, or done [11] and are unable to question their own judgement [12]. One increasing interest is that these problems may extend to difficulties examining one’s own behaviors, thoughts, and feelings and analyzing one’s ideas about others [13,14]. Metacognition is divided into many dimensions of ”thinking about thinking”, including explicit awareness about knowledge (for example, knowing that I feel tense in a crowded room or that my memory is fallible) and implicit awareness (for example, knowing that I should breathe slowly when I am stressed or when a task is difficult and that I should work slowly) [15].

In all the studies we covered in this paper, different subcomponents of metacognition and their specific interactions were considered. The subcomponents that researchers studied varied from social cognition [13] to metamemory [16,17,18]. One of the notable interactions was the interaction between metacognition and work performance [19,20] (Table 1).

Comparative studies were also addressed between schizophrenia spectrum disorders and different psychiatric disorders, predominantly bipolar disorder [26,32]. A comparison based on symptoms experienced by schizophrenia patients was carried out by Bruno et al. the main symptom targeted being the presence of delusional ideas, which are maintained by metacognitive deficits. Over the past years, researchers have made efforts to validate the new scales, one such initiative being that of Brocker and his team [37,71].

The reasons for carrying out the study in the field of metacognition on schizophrenia is varied according to the purpose defined by each research team, although patterns for the association between different positive or negative symptoms and metacognition are often encountered. For example, Luther et al. [32] wanted to see if metacognition is necessary for motivation to occur in participants with schizophrenia or a schizoaffective disorder; Lysaker et al. [38], wanted to test the results obtained previously by other research teams [22,72] but on a group of Spanish-speaking subjects from Chile. Those studies demonstrate that, in general, people with schizophrenia have more pronounced cognitive impairments than bipolar patients, and that cognition is associated with the severity and frequency of the negative symptoms [38,40].

Moritz and Woodward wanted to determine whether knowledge corruption is present in patients with schizophrenia (holding false information with the belief that it is true, representing a metamemory defect), and a comparison between schizophrenia patients, OCD patients, and PTSD patients according to this aspect. Moreover, they wanted to see whether this impairment is less pronounced in schizophrenia than in OCD or PTSD and if there is a link between hallucinations and misattribution of self-generated negative information to external sources [42].

In their study on metacognition, Minor and Lysaker also considered a series of symptoms specific to schizophrenia, namely disorganized symptoms (thinking, speaking, etc.) and wanted to identify whether they are inversely correlated with cognitive processes and if they successfully moderate the relationship between neurocognition, social cognition, and metacognition [45].

The aim of the research conducted by Nicolo et al. was to replicate the results of the study conducted by Lysaker in 2005, but on Italian adults with schizophrenia. The parameters measured in this research were three components of metacognition, namely self-awareness, awareness of the thoughts of others (awareness of the mind of others), and mastery. They were correlated with positive and negative symptoms, depression level, knowledge, and five domains of neurocognition [22,50].

Bonfils and his team tested the assumption that high metacognition levels and reduced stress would be associated with improved performance in cognitive empathy and affective empathy in people with schizophrenia spectrum disorders [50,55]. Moreover, they wanted to see if metacognition moderates the relationship between distress and empathy in the case of the same group of patients [50].

Wright et al. [65] wanted to see if reduced expectations regarding metacognition at a given moment would be associated with the presence of hallucinations and whether the severity, intensity, duration, valence, and degree of distress that the hallucinations generate might influence patients’ expectations about their metacognitive capacity. Another aim of the research was to demonstrate whether initial and final evaluations of metacognition and emotion recognition are closely related and whether deficits in metacognition and emotion recognition are possibly associated with the presence of negative symptoms and disorganized thinking, assessed both concurrently as well as prospectively [65]. Hamm et al. wanted to observe if there is a relationship between emotion recognition and three sets of cognitive domains, namely theory of mind (the ability to understand people by attributing mental states), metacognition, and neurocognition [61].

The number of participants included in the studies covered ranged between 21 and 175, this aspect being influenced by the aim of each research team. Studies carried out by Luther et al. or Lysaker et al. had a larger number of participants, 175 [38] and 173 respectively [62], for their previously described objectives. As for the studies that involved comparisons between schizophrenia spectrum disorders and other psychiatric conditions, the participant number was well-defined for each group: 31 participants with schizophrenia or schizophreniform disorder, 20 participants with OCD (obsessive–compulsive disorder) and 28 participants with diagnosed PTSD in one study [42], 26 schizophrenia patients, 26 bipolar patients, and 36 healthy controls in another [40].

Additionally, another essential aspect of the studies included was the exclusion criteria of the patients, which were:

√ Epilepsy
√ Various neurological diseases
√ Cognitive deficiencies
√ Medication changes
√ Cranial trauma
√ Delirium
√ Hospitalizations
√ Substance abuse

After analyzing the available data from the studies included, which aimed the analysis of the different subcomponents of metacognition, but also the interactions between them, we can say that certain subcomponents of metacognition are associated with social cognition. Lysaker et al. showed metacognition is strongly associated with social cognition and work performance [19].

The most studied subcomponent, however, was metamemory. Bacon and Izaute showed that metamemory is strongly reduced in schizophrenia and it cannot be improved even by regular memory exercises, with no differences between patients who do these exercises and those who do not [16]. As for the interaction between metacognition and work performance, studies have shown that patients with a high level of metacognition perform better on tasks at work [19,20].

Comparative studies carried out by researchers have shown that patients with schizophrenia have a low level of metacognition, which means they have difficulties in forming complex ideas about themselves and the world and also impaired social function [24]. Tas et al. observed that patients with schizophrenia have lower metacognitive abilities than bipolar patients [30]. The comparative study carried out by Bruno et al. showed that schizophrenia patients who had delusions also had metacognitive deficits [35]. An effort of Brocker et al. to validate the MAS-A (Metacognition Assesment Scale Abbreviated) scale was successful, and the results obtained are similar to those in the literature [73].

**Figure 1:**
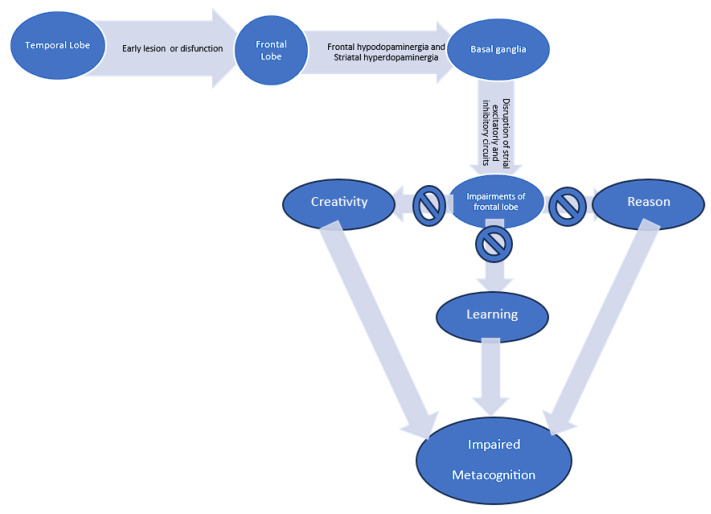
Pathophysiology of schizophrenia and the relationship to metacognition [74].

Metacognition as a cognitive process has been studied in different contexts to determine the different interactions that it may have with other cognition domains or schizophrenia symptoms (Figure 1).

In this way, Hamm et al. (2012) [61], showed that metacognition and social cognition assessments in patients with schizophrenia or schizoaffective disorder are stable over time. The results also showed that metacognition is associated with subsequent symptom assessments. Regarding symptoms, as hypothesized, baseline ratings of metacognition were significantly correlated with severity of negative symptoms and disorganized thinking measured with the PANSS (Positive and Negative Syndrome Scale), both concurrently and prospectively [61].

Contrary to expectations, baseline emotion recognition performance was only related to disorganized thinking at both assessment time points, with a significant relationship to negative symptoms only at baseline. Emotion recognition performance was also related to positive symptom severity but only at follow-up [61]. Another group of researchers showed that patients diagnosed with a schizophrenia spectrum disorder performed less well than the control group in terms of emotion recognition, mental state decoding, mental state reasoning, and metacognition. All of these components were independently related to emotion recognition within patients with schizophrenia or schizoaffective disorder. Moreover, the results revealed that impaired emotion recognition in schizophrenia may result in part from a combination of difficulties in the ability to judge the cognitive and affective states of others as well as in creating representations of self and others [62].

Metacognition is relevant when we are talking about positive symptoms (such as hallucinations). Wright et al. showed that metacognition deficits were associated with a higher number of hallucinations, but a less accurate completion probability and standard was associated with a lower number of hallucinations [65]. Another study regarding metacognition in schizophrenia spectrum disorders showed that metacognition is positively and significantly associated with performance on tasks that measure cognitive empathy and affective empathy, and this result is valid for all 4 subscales of the MAS-A (self-awareness; awareness of others’ mental processes; decentering; mastery) [55].

Contrary to initial expectations, the results also revealed that distress is not associated with empathy [48]. It was also demonstrated that metacognition moderated the relationship between distress and performance on tasks measuring affective empathy, but not the relationship between distress and performance on tasks measuring cognitive empathy. In fact, increased distress improved performance on tasks measuring affective empathy for those with a low level of metacognition and reduced affective empathy performance for those with a higher level of metacognition [55]. Mediavilla et al. showed that schizophrenia patients had fewer autobiographical memories compared to healthy subjects, but more semantic associations. The researchers’ results demonstrated that metacognition mediates the relationship between schizophrenia and autobiographical memory [57].

Metacognition’s influence in the life of schizophrenia patients is also relevant when we are talking about motivation. A study carried out by Luther et al. showed that minimal levels of metacognition do not necessarily facilitate high motivation, mainly because patients with sub-minimum levels cannot have high motivation [38]. Another study that assessed intrinsic motivation in schizophrenia showed that high metacognitive mastery scores had greater value for intrinsic motivation than medium or low scores [55]. However, there were no differences between patients in terms of intrinsic motivation [55]. A final study that assessed a range of negative symptoms and their interaction with metacognition was conducted by Minor and Lysaker. They demonstrated that symptoms within the “disorganization” symptom cluster had significant inverse relationships to those schizophrenia-specific symptoms targeted in the study, the association being much more significant between metacognition and disorganized symptoms. Disorganized symptoms modulate neurocognition, social cognition and metacognition [45].

The metamemory subcomponent is often addressed, and a notable study is that of Moritz and Woodward, in which they showed that schizophrenia patients show similar metamemory deficits in comparison with healthy subjects, PTSD patients and OCD patients. However, knowledge corruption was more pronounced in schizophrenia than in posttraumatic stress disorder or obsessive–compulsive disorder [42]. The authors attributed those results to the overconfidence schizophrenia patients had in incorrect answers and underconfidence in correct answers (most likely due to their already-existing cognitive deficiencies) [42]. Another study on metamemory showed that only some aspects of metamemory were related to self-perception, with participants scoring higher in memory-related anxiety. They had lower confidence in their use of problem-solving strategies, mental capacities, and memory abilities. However, basic memory processes, motivation to succeed, and perception of aging were similar to healthy individuals [51].

The approach to studying metacognition at a global level was also continued by Nicolo et al. (2012) [50] following the study carried out by Lysaker (2005) [22]. Nicolo’s team managed to replicate the results previously obtained by Lysaker. In accordance with what Lysaker previously obtained, the synthetic functions of metacognition were associated with negative symptoms and neurocognition, a parameter of perspicacity that was closely related to the ability of the individual to reflect on one’s own thoughts. An aspect that was not replicated, however, tests the link between metacognition, positive symptoms, and emotional discomfort [22,50]. Another replication with a group of Spanish-speaking participants from Chile had results similar to those of the English study group when schizophrenia patients were compared to bipolar patients and healthy participants. Therein, schizophrenia patients had lower metacognition levels and a greater preponderance of symptoms as well as a higher degree of severity [40].

## 4. Discussion

Our review revealed that many patients with schizophrenia experience metacognitive deficits or impaired metacognitive abilities [30]. In the study conducted by Popolo and his team, there were differences regarding the age of the participants, their level of education, and their symptoms. The number of men and women taking part in the study was disproportional, but still limited to 72 (26 schizophrenia patients, 23 bipolar patients, and 23 healthy controls). Even so, the study has demonstrated that metacognitive deficits lead to impaired social function [24] and are strongly associated with poorer self-awareness and anosognozia, as shown in literature [9,10].

Hamm et al. demonstrated that metacognition and social cognition are stable over time. Significant correlations were found between the before-mentioned cognition domains and the severity of symptoms at baseline and 6 months later. Moreover, disorganized symptoms exhibited significant negative associations with cognitive processes [61]. Minor and Lysaker obtained similar results, thus disorganized symptoms were inversely associated with cognitive processes and modulated social cognition and neurocognition. No significant relationship was found between negative symptoms and cognitive processes [45]. Lysaker et al. as well as Nicolo et al. observed strong correlations between negative symptoms, metacognition and neurocognition [22,50].

Regarding positive symptoms, Wright et al. [65] showed that schizophrenia patients who had metacognitive deficits were also likely to experience more hallucinations. Surprisingly, those patients who had a less accurate completion probability and standard had a lower number of hallucinations [65]. Hallucinations were also associated with metacognitive deficits and emotional discomfort, as shown by Lysaker [22]. When compared with bipolar patients and healthy subjects, patients diagnosed with a schizophrenia spectrum disorder had a lower level of metacognition, which in turn was associated with more frequent, more severe symptoms [40].

Metamemory, a subcomponent of metacognition, and memory deficits were also addressed. Moritz and Woodward observed no difference between schizophrenia patients, OCD patients, and PTSD patients in terms of metamemory deficits. However, knowledge corruption was specifically impaired in those with schizophrenia [42]. Bacon and Izaute [16] made a computerized version of the FOK test, obtaining major differences regarding short-term memory between the control group and the group of participants with schizophrenia. They attributed these results to the cognitive deficits already present in schizophrenia patients, the relatively small group, and the differences between participants regarding knowledge. It was also shown that schizophrenia patients have fewer autobiographical memories than healthy individuals [57].

Regarding motivation in patients diagnosed with a schizophrenia spectrum disorder, Luther et al. showed via NCA (Necessary Condition Analysis) that a certain metacognition level is essential for motivation to occur. The results also showed that motivation cannot occur in participants with below-average metacognition scores [38]. Those results were similar to those obtained previously by Vohs and Lysaker, who demonstrated that metacognitive mastery is associated with intrinsic motivation [54]. A major limitation of the study conducted by Luther et al. is that most of the participants were men receiving outpatient services. In this case, the generalizability of the study is limited [38].

Metacognition is also essential for better work performance and improved social cognition. For example, Lysaker and his collaborators conducted a study in which they divided the study participants in two treatment groups, one in which participants received cognitive behavioral therapy and one in which the participants received support at their workplace. The participants had an hourly job for 26 weeks in an entry-level position. Their evaluation was conducted twice a week with WBI without the researchers knowing their IPII scores. The results showed that patients high in self-reflectivity (a domain of metacognition) had better work performance than patients with medium or low scores. It should be mentioned that during the statistical analysis, there were discontinuities in the data because they was not constant, weekly interpolation being necessary [19].

For the comparative studies involving participants with schizophrenia and bipolar disorder, it is worth mentioning that the results can be explained by the nature of the disorders themselves. Although those two psychiatric conditions share some symptoms (such as delusional ideas, disorganized speech, anosognosia, and in some cases depressive symptoms), the way they manifest is completely different. Patients with bipolar disorder have periods of time when mood is elevated, they are predominantly euphoric, and they have high energy levels and racing thoughts (the so-called manic episodes) and periods of time when they have a predominantly depressive mood, lack of energy, anhedonia, avolition, and decreased/increased appetite (or the so-called depressive episodes). Instead, when we are talking about schizophrenia, we are talking about a persistent affective flattening, the presence of hallucinations of any kind, delusional ideas, and in many cases cognitive deficits [30,38,40].

It is still important to note that different treatment additions, such as activities completed as part of a treatment plan, are beneficial, but those changes can also be replicated by the addition of different adjuvant substances that can play a crucial role, such as in treating depression in schizophrenia patients. However, it should be noted that compared to the study of metacognition in schizophrenia, the additions of antipsychotic treatments to various activities and adjuvant medications currently require a more extensive understanding [19,71,75].

Finally, regarding selection criteria and number of the participants, we can say that they were different for each study group. For the study conducted by Popolo and his team, for example, there were major discrepancies in terms of age, gender, level of education, and symptoms of the participants diagnosed with schizophrenia or bipolar disorder. As we mentioned before, the number of participants in this study was limited to 72 [24].

Tas et al. [30]. had instead a different way of choosing their participants. They chose patients in the residual phase of schizophrenia and compared them with bipolar patients. It should be mentioned that the application of the MAS-A scale for participants with psychosis took longer than for the participants who did not have psychosis. Bruno et al. [35] conducted the study using a computerized, modified form of the WCST [28], but it was not specified how the changes made to the original test influenced the results. The number of participants in this study was also limited [35].

## 5. Conclusions

Schizophrenia is a serious psychiatric condition that negatively influences the quality of life not only of those receiving this diagnosis but also of their family members. Practically speaking, people affected by schizophrenia have a lower work performance, lower motivation, and significant cognitive deficits. They also have a poorer self-awareness and awareness of others, which are the main aspects of metacognition.

To date, studies have shown that metacognition influences work performance, autobiographical memory, motivation, the severity of symptoms, and social cognition and emphasized how schizophrenia symptoms interact with metacognition, which represents mental health. Future studies with more participants are needed in order to form a more complex image about metacognitive deficits in schizophrenia and the way they interact with certain aspects of life.

## Figures and Tables

**Table 1:** Metacognition-related aspects in the pathology of schizophrenia.

Authors	Approached Metacognition Component	Instruments	Results
[13]	Social cognition and metacognition	IPII [21], MAS-A [22]	Lysaker et al. showed that some aspects of social cognition are associated with metacognition.
[16]	Metamemory	FOK [17,18,23]	Recall of the letter sequences in patients with schizophrenia was significantly reduced.
[24]	Motivation and metacognition	MAS-A [22], IPII [21], MCQ-30 [25], BPRS [26,27]	The results indicated that patients with schizophrenia were less able to form complex ideas about themselves or others and use metacognitive knowledge.
[19]	Work performance and metacognition	MAS-A [22], IPII [21], WCST [28], WBI [29]	The results showed that indeed, patients with schizophrenia who have high levels of self-reflectivity also have improved working performance.
[30]	Metacognition	PANSS [31], HDRS [32], YMRS [33], WMS-III [34], WCST [28], MAS-A [22], IPII [21]	Schizophrenia patients had a lower capacity for self-awareness compared to bipolar patients.
[35]	Delusions and metacognition	SANS and SAPS [36], BCIS [37]	The results obtained by Brunno et al. showed that indeed, all schizophrenia patients have metacognitive deficits, but this association is more prominent in schizophrenia patients with delusions.
[38]	The level of metacognition required for the emergence of motivation	MAS-A [22]; IPII [21]; QLS [39]	Statistical analysis results demonstrated that metacognition is required for motivation to occur in patients with a diagnosis of schizophrenia or schizoaffective disorder.
[40]	Magnitude of metacognitive deficiencies in various mental disorders	IPII [21]; MAS-A [22]; IRI [41]; PANSS [31]	Schizophrenia patients showed lower metacognition, higher symptom prevalence, and higher severity compared to bipolar patients.
[42]	Metamemory in schizophrenia	Y-BOCS [43]; PANSS [31]; PANADSS [44]	Patients with schizophrenia have more significant deficiencies in metamemory compared to control groups (healthy subjects, PTSD subjects, OCD subjects).
[45]	A series of symptoms correlated with social cognition, metacognition, and neurocognition	PANSS [31]; MATRICS [46]; MSCEIT [47]; BLERT [48]; SAT-MC [49]; IPII [21]; MAS-A [22];	The symptoms present in the group of symptoms “disorganization” were correlated negatively but significantly with neurocognition, social cognition, and metacognition.
[50]	Correlation of metacognitive capacity with perspicacity and neurocognitive deficits	PANSS [31]; IPII [21]; MAS-A [22];	The synthetic aspects of metacognition (especially disease insight) have been associated with negative symptoms and neurocognition.
[51]	Metamemory and knowledge confidence in patients with schizophrenia	MIA [52]; WAIS (WAIS-R; [34]; WMS-R [53];	Memory performance and overall IQ were lower among schizophrenia patients.
[54]	Metacognitive mastery and intrinsic motivation	QLS [39]; PANSS [33]; IPII [21]; MAS-A [22];	High scores for the “mastery” subscale had a higher value for intrinsic motivation compared to average or low scores.
[55]	Metacognition mediates the relationship between anxiety and empathy	Derntl paradigm [56]; MAS-A [22]; IPII [21]; IRI [43]; PANSS [31];	Metacognition is positively and significantly associated with performance on tasks that measured cognitive empathy and affective empathy in people with schizophrenia or schizoaffective disorders.
[57]	Autobiographical memory and metacognition	MAI [58]; MAS-A [22]; AMT [59]; SCIP [60];	The findings showed that patients with schizophrenia had fewer autobiographical memories than healthy subjects, but more semantic associations.
[61]	Metacognition and social cognition. Stability of symptoms over time	IPII [21]; MAS-A [22]; BLERT [48]; PANSS [31]; WCST [28]	Results have shown that assessments of metacognition and social cognition in schizophrenia or schizoaffective patients are stable over time.
[62]	Metacognition, neurocognitive functions, and emotion recognition	BLERT [48]; Eyes Test [63]; The Hinting test [64]; IPII [21] MAS-A [22]; PANSS [31]; WCST [28]	Patients with schizophrenia were less successful than controls in recognizing emotions, decoding mental status, and engaging in mental state reasoning and metacognition.
[65]	Poorer momentary metacognitive expectancies associated with hallucinations	MUSEQ [66]; CAPS [67]; CDS [68]; MSAS [69]; BCIS [27]; SENS [70];	The results showed that a lower level of metacognition was associated with a higher number of hallucinations, but the probability and less accurate standard of completion were associated with a lower number of hallucinations.

IPII—Indiana Psychiatric Illness Interview; BLERT—The Bell-Lysaker Emotional Recognition Task; PANSS—Positive and Negative Syndrome Scale; WCST—Wisconsin Card Sorting Test; MAS-A—Metacognition Assessment Scale-Abbreviated; Eyes Test—Reading the Mind in the Eyes Test; FOK—Feeling of knowing; MCQ-30—Metacognitions Questionnaire-30; MAS—The Metacognition Assessment Scale; WBI—Work Behavior Inventory; HDRS—Hamilton Depression Scale; YMRS—Young Mania Rating Scale; WMS III—Wechsler Memory Scale, Third Edition; BCIS—Beck Cognitive Insight Scale; SANS—The Scale for the Assessment of Negative Symptoms; SAPS—Simplified Acute Physiology Score; QLS—Quality of Life Scale; IRI—Interpersonal Reactivity Index; Y-BOCS—Yale–Brown Obsessive–Compulsive Scale; PANADSS—Positive and Negative and Disorganised Symptoms Scale; MATRICS—The Measurement and Treatment Research to Improve Cognition in Schizophrenia; MSCEIT—Mayer–Salovey–Caruso Emotional Intelligence Test; SAT-MC—Social Attributions Test— Multiple Choice; MIA—Metamemory Inventory in Adulthood; WAIS-R—Wechsler Adult Intelligence Scale-Revised; WMS-R—Wechsler Memory Scale-Revised; MAI—Metacognitive Assessment Interview; SCIP—Screen for Cognitive Impairment in Psychiatry; AMT—Autobiographical Memory Test; MUSEQ—Multimodal Unusual Sensory Experiences Questionnaire; CAPS—Cardiff anomalous perceptions scale; CDS—Cambridge depersonalization scale; MSAS—Metacognitive Self-Assessment Scale; SENS—Self-Evaluation of Negative Symptoms.

## Data Availability

All data is available on request.
